# *Pseudomonas aeruginosa* outcompetes other bacteria in the manifestation and maintenance of a biofilm in polyvinylchloride tubing as used in dental devices

**DOI:** 10.1007/s00203-016-1208-6

**Published:** 2016-03-15

**Authors:** Christoph Gert Ammann, Markus Nagl, Michael Nogler, Débora Cristina Coraça-Huber

**Affiliations:** Experimental Orthopaedics, Medical University of Innsbruck, Innrain 36 - 1. Floor, 6020 Innsbruck, Austria; Division of Hygiene and Medical Microbiology, Department of Hygiene, Microbiology and Social Medicine, Medical University of Innsbruck, Schöpfstrasse. 41, 6020 Innsbruck, Austria

**Keywords:** Bacterial biofilm, PVC tubing, Dental device, Co-culture

## Abstract

In a PVC tube as a model system for dental devices, *Pseudomonas aeruginosa* outcompetes *Staphylococcus aureus* and *Klebsiella pneumoniae* for the biofilm formation*. P. aeruginosa* has advantage over the other strains due to higher tolerance for low-nutrient situations or direct killing by the production of soluble factors like pyocyanin.

## Findings

Polyvinylchloride (PVC) tubes are widely used in medical and dental devices. These tubes can easily come in contact with human skin and mucosa during odontology procedures and can be contaminated with the following bacteria that play a role in human medicine.

*Pseudomonas aeruginosa* is a Gram-negative rod-shaped bacterium found in moist to wet habitats. These include human mucosal surfaces, e.g., the nasopharynx (Fothergill et al. 2014), and surfaces in tap water lines (Rozej et al. 2015). *Klebsiella pneumoniae* is a Gram-negative rod-shaped bacterium that can colonize mouth mucosal tissue (Bagley 1985) and cause pneumonia (Podschun and Ullmann 1998). *Staphylococcus aureus* is a Gram-positive bacterium that typically colonizes skin. Approximately 20 % of all human beings are long-term carriers of *S. aureus* in nose mucosa (Kluytmans et al. 1997).

Once in contact with the moist surface of a tube lumen, bacteria can adhere to the material and start biofilm formation. Biofilms are formed in several stages during propagation of bacteria after adherence (Stoodley et al. 2002). Once a sufficient number of bacteria are reached, the biofilm matures, ultimately establishing an extracellular matrix (ECM). The ECM contains water, polyglycans, proteins, and nucleotides (Branda et al. 2005). Bacteria in the biofilm use these stored materials in times of malnutrition from the exterior.

We here investigate the growth of three bacterial strains which are typical components of human flora. These can transfer from human to a dental device and subsequently grow in the lumen of the PVC tubing. We sought to determine the growth pattern and dynamics of *S. aureus* and *K. pneumoniae* in co-culture with *P. aeruginosa*, a well-known contaminant of water systems.

*Pseudomonas aeruginosa* ATCC 27853, *S. aureus* ATCC 25923, and *K. pneumoniae* (clinical isolate) single colonies from Mueller–Hinton (MH) agar were grown separately overnight at 37 °C in MH broth to 2–5 × 10^9^ colony-forming units (cfu)/mL. Subsequently, *P. aeruginosa* was mixed with *S. aureus* or *K. pneumoniae* and diluted in MH broth to approximately 1 × 10^5^ cfu/ml for each strain. One tube system each was filled with *P. aeruginosa* plus *S. aureus* or *P. aeruginosa* plus *K. pneumoniae*. We here sought to speed up the process of initial biofilm formation by providing ample nutrients (MH broth) for 72 h. After this period, we provided tap water for the system and followed the bacterial count of the formed biofilms for 5–8 weeks. To imitate a dental device system, we cultivated the biofilm at room temperature and provided flow of water by a peristaltic pump.

**Fig. 1:**
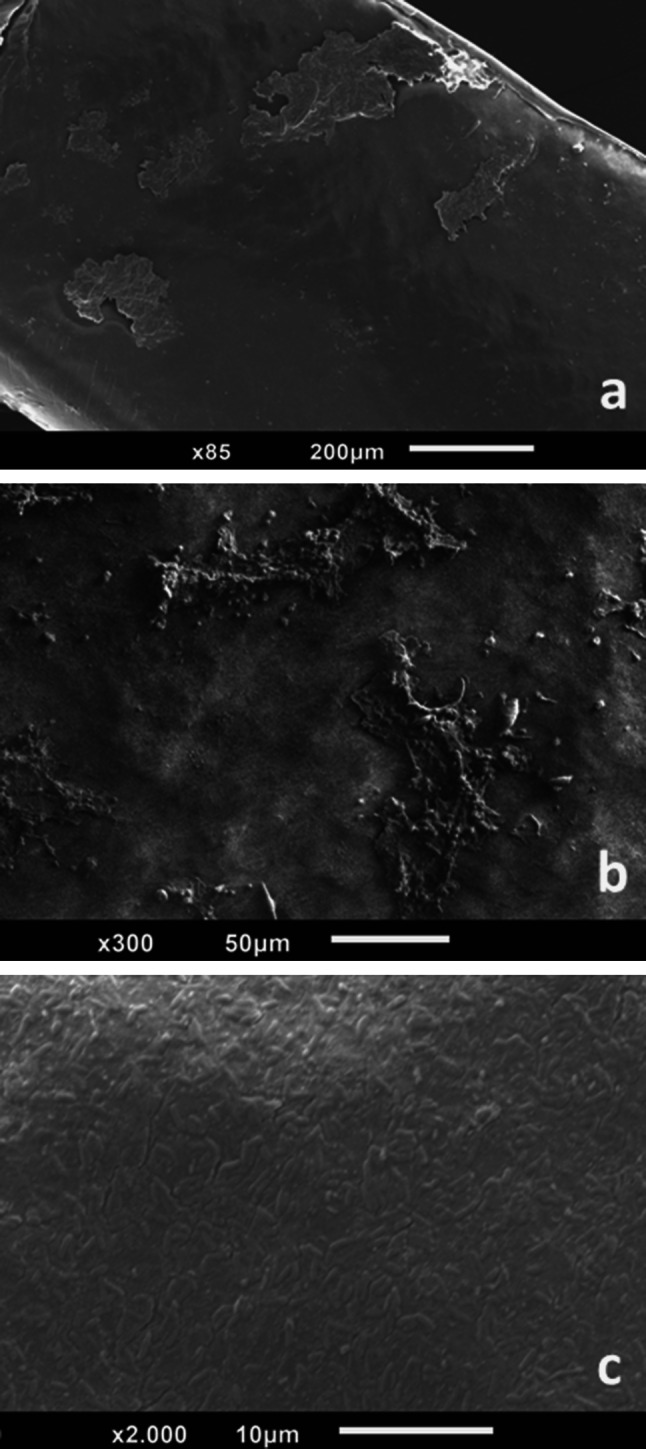
Scanning electron microscopy image of bacterial biofilms spread over the PVC tube lumen. **a** Small areas of biofilms spread on the PVC tube lumen; **b** *Staphylococcus aureus* biofilm on PVC lumen; **c** PVC lumen populated by *Pseudomonas aeruginosa* biofilm

We used scanning electron microscopy to visualize the biofilms on the tube lumen and collect data on their spatial distribution at the end of the incubation period. After fixation and dehydration, samples were investigated in a scanning electron microscope (Jeol 6010, Eching, Germany) at 5 kV acceleration voltage using a spot size of 40 or 50, respectively. We used the REF detector setting in which data are collected through the secondary electron detector without using the suction current to attract secondary electrons. We found that biofilms are clustered in small groups of high bacterial counts instead of forming a single low-density biofilm spread over the entire tube lumen (Fig. 1a, b). Similar results have been observed with confocal microscopy using ground water and no specific bacterial input (Martiny et al. 2003).

**Fig. 2:**
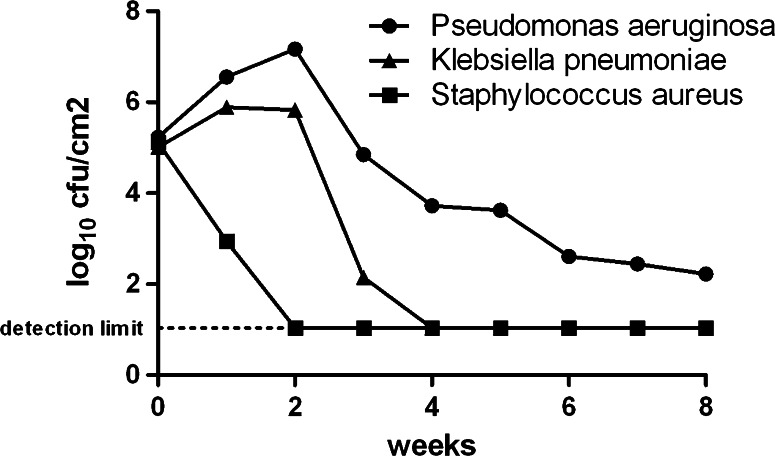
Bacterial counts per square centimeter of tubing; mean values of *n* = 2 for *P. aeruginosa* (*filled circle*), single values for *K. pneumoniae* (*filled triangle*) and *S. aureus* (*filled square*)

The single biofilms showed a thick ECM spreading over the biofilm core, while single bacteria could still be visualized at the edges of the individual biofilms. A representative image is shown in Fig. 1b. At weekly intervals, a sample of the PVC tube was cut and stained with 0.5 % crystal violet for 5 min for macroscopic evaluation of the biofilm growth. We observed a gradual covering of the lumen along the time beginning with isolated clumps. Additionally, we weekly cut a 10-cm piece of tubing. 3 cm were cut from the non-pump side, washed extensively, and placed in 6 ml of MH medium. The biofilm was then detached from the tube by ultrasound (41 kHz, Bandelin Bactosonic, Berlin, Germany) for 1 min. Bacterial counts were determined after transferring 100 µl of the solution to a MH agar plate leading to a limit of detection of 10 cfu/ml. At end, we calculated how many bacteria were attached to the tube lumen per square centimeter. Figure 2 shows the determined values for both experimental settings combined.

*Pseudomonas aeruginosa* proved to be the predominant bacterial strain overgrowing both *S. aureus* and *K. pneumoniae*. All strains were initially detected at a similar level of approximately 10^5^ cfu/cm^2^. After only 2 weeks, *S. aureus* had vanished from the system, while *P. aeruginosa* showed still increasing colony counts. *K. pneumoniae*, after slightly increasing from 5.01 to 5.83–5.89 log_10_ cfu/cm^2^ during the initial 2 weeks, decreased rapidly in the third week and was undetectable at any time point in the following weeks. The overgrowth by *P. aeruginosa* may either be an indirect effect of this species being better adapted to low-nutrient situations, or it may reflect a direct killing of the partners in co-culture by secreted toxins like pyocyanin (Voggu et al. 2006). In contrast to the vanishing of *S. aureus* or *K. pneumoniae*, the cfu counts of *P. aeruginosa* plateaued rather stably at approximately 200 cfu/cm^2^ of tube lumen from week six on. This is in agreement with early reports showing that in dental unit water lines shedding of 10^2^ to 10^6^ cfu/ml can occur (Gross et al. 1976; Furuhashi and Miyamae 1985).

Our data show that biofilms can be formed and maintained in tap water on the lumen of PVC tubes using the method developed here. Pseudomonas outcompeted the respective partners in co-cultures and established long-lived biofilms in water according to our experiments. The long-time incubation of *P. aeruginosa* with water has recently been shown to alter the phenotype significantly (Mendis et al. 2014). Our future experiments will focus on the genotypic and resulting phenotypic changes in long-term dental unit biofilms as well as the sterilization of biofilms with novel agents.
